# High-Altitude Condition Induces Hepatic Magnetic Susceptibility Changes and Liver Injury

**DOI:** 10.3390/biom16030353

**Published:** 2026-02-26

**Authors:** Xiaoyuan Zhou, Chuanlin Feng, Jingming Fu, Lei Zhang, Chao Song, Junjun Wang, Lin Chen, Xin Zhang

**Affiliations:** 1 Institutes of Physical Science and Information Technology, Anhui University, Hefei 230031, China; q23301421@stu.ahu.edu.cn (X.Z.); q23301398@stu.ahu.edu.cn (J.F.); 2 Chinese Academy of Sciences High Magnetic Field Laboratory, Hefei Institutes of Physical Science, Hefei 230031, China; fcl@mail.ustc.edu.cn (C.F.); leizhang@hmfl.ac.cn (L.Z.); chaosong@hmfl.ac.cn (C.S.); junjunwang1222@hmfl.ac.cn (J.W.); 3 Science Island Branch of Graduate School, University of Science and Technology of China, Hefei 230031, China

**Keywords:** high altitude, iron metabolism, magnetic properties, magnetic susceptibility, liver injury

## Abstract

Millions of people reside in hypobaric, hypoxic high-altitude environments, yet the chronic consequences of sustained exposure remain incompletely understood. Liver magnetic resonance imaging (MRI) in residents at different altitudes revealed signal alterations suggestive of changes in magnetic susceptibility and tissue composition. To further investigate these observations, mice were exposed to simulated 5000 m hypobaric hypoxia for six weeks. High-altitude-exposed mice developed significant liver impairment, characterized by increased hepatocyte apoptosis and elevated magnetic susceptibility. Quantitative analyses demonstrated approximately a two-fold increase in hepatic iron content accompanied by the formation of iron aggregates. Concomitant lipid accumulation and oxidative stress were observed, indicating coordinated disruption of iron homeostasis and metabolic balance. Collectively, these findings suggest that high-altitude-associated iron accumulation contributes to magnetic susceptibility alterations and promotes liver injury through dysregulated lipid metabolism and oxidative stress, providing mechanistic insight and potential implications for high-altitude risk assessment and clinical management.

## 1. Introduction

High-altitude regions (≥2500 m above sea level) attract millions of visitors each year and are home to over 80 million permanent residents worldwide [1]. Astronauts during critical space mission phases, such as extravehicular activities, are also exposed to similar or even worse conditions. In such environments, the reduced partial pressure of oxygen induces systemic hypoxia [1,2]. One of the important regulatory mechanisms mammals use to adapt to high-altitude hypoxia is compensatory erythrocytosis, which increases the number of hemoglobin-containing red blood cells capable of transporting oxygen [3]. Since iron is essential for hemoglobin synthesis [4] and the liver serves as the central organ for iron storage and metabolism [5], we hypothesize that the liver plays a critical role in high-altitude acclimatization. However, whether and how high-altitude conditions influence hepatic iron homeostasis remain unclear.

Iron metabolism is tightly linked to liver function. For example, hepatocyte iron deficiency has been shown to promote lipogenesis and insulin resistance, whereas iron overload stimulates reactive oxygen species (ROS) production and fibrogenic activation in hepatic stellate cells [6]. As a highly metabolic organ with substantial energy demands [7], the liver is particularly vulnerable to functional impairment under energy-limited conditions such as hypoxia. Nevertheless, the effects of high-altitude exposure on liver physiology remain poorly understood and lack systematic investigations.

Beyond its role in hemoglobin synthesis, iron is a key determinant of tissue magnetic properties, as paramagnetic iron can induce local magnetic field perturbations in the presence of an external magnetic field. These susceptibility-related effects can be detected using magnetic resonance imaging (MRI) [8,9,10]. Given its high spatial resolution and non-invasive nature, MRI is widely used to characterize pathological changes in various tissues, including the nervous and musculoskeletal systems [11]. Chemical shift-based MRI, such as dual-echo in-phase and out-of-phase imaging, reflects differences in signal behavior arising from water–fat interactions in T2* decay [12]. In these sequences, in-phase images are typically acquired over a longer echo time than out-of-phase images, allowing greater time for signal decay. As paramagnetic substances such as iron accelerate local dephasing and shorten T2*, increased iron deposition may manifest as reduced signal intensity, appearing darker on MRI, whereas relatively preserved signal appears. These strengths make MRI well suited for probing iron-related alterations in hepatic tissue under hypoxic stress.

In this study, we integrated MRI data from high-altitude residents with a mouse model of hypobaric hypoxia simulating 5000 m to investigate liver injury and associated changes in magnetic property. We demonstrate that high-altitude exposure induces hepatic iron overload, leading to markedly elevated liver magnetization. These changes occur alongside a spectrum of pathological features, including lipid accumulation, oxidative stress, apoptosis, and inflammation. Our findings provide mechanistic insights into preventing high-altitude-associated liver injury and advance the understanding of magnetic biological effects in the liver under hypoxic conditions.

## 2. Materials and Methods

### 2.1. Participants and Liver MRI Protocol

Seven volunteers (6 males and 1 female, 40–67 years old) were recruited from regions of various altitudes (from <50 m to 4500 m) to capture altitude-associated variation in liver magnetic susceptibility. All participants were permanent residents who had lived at their respective altitudes. The research was approved by the Research Ethics Board at Qinghai Provincial People’s Hospital, Qinghai University (Ethical number: (2024)-024-03) and was conducted in accordance with the 1964 Helsinki Declaration and its later amendments or comparable ethical standards. Informed oral consent was obtained from all the participants of the study.

Liver MRI images were acquired using a 1.5 T MRI scanner (Signa HDxt, General Electric Healthcare, Waukesha, WI, USA) with an 8-channel phased-array body coil. Participants fasted ≥4 h before scanning to reduce postprandial changes in hepatic iron and fat content. Participants were positioned supine with arms raised, and the receive coil was centered at the xiphoid process to align the liver with the isocenter of the magnet. Respiratory motion was minimized by placing a gating sensor at the chest–abdomen junction, and data were acquired during end-expiratory breath-holds. A T1 dual-echo in-phase/out-of-phase sequence was used to evaluate susceptibility-related signal changes. Parameters included: repetition time (TR) = 180 ms, TE = 2.2 ms (out-of-phase) and 4.2 ms (in-phase), flip angle = 80°, field of view (FOV) = 420 × 420 mm^2^, slice thickness = 8 mm, and interslice gap = 2 mm. Liver function tests and routine hematology were obtained within 24 h of MRI, except in one participant who did not undergo liver function testing.

### 2.2. Mouse Model of High-Altitude Hypoxia and Physiological–Behavioral Assessment

All animal experiments followed the International Guiding Principles for Biomedical Research Involving Animals and were approved by the Animal Care and Use Committee of the Hefei Institute of Physical Science, Chinese Academy of Sciences (DWLL(E)-2024-36). Balb/c mice (Nanjing GemPharmatech, Nanjing, China) were acclimated for one week under specific pathogen-free conditions (22–24 °C, 60% ± 10% humidity, 12 h light/dark cycle). Mice were randomly assigned to either a normoxia control group or a high-altitude group, with each group consisting of ten male mice, housed in a hypobaric hypoxic chamber simulating 5000 m for six weeks to establish a high-altitude mouse model. All other conditions were the same for all mice, other than the high-altitude simulation. The chamber continuously adjusts its pressure and oxygen levels to replicate low-pressure, hypoxic high-altitude environments [13].

Physiological monitoring

Physiological parameters were recorded in conscious mice using the MouseOx system (Starr, Oakmont, PA, USA). Each mouse was placed in a transparent chamber for 10 min to measure arterial oxygen saturation, heart rate, respiratory rate, and pulse distension. The chamber was disinfected with 75% ethanol and dried between trials, and all experiments were conducted between 9:00 a.m. and 5:00 p.m.

Behavioral assessment

To evaluate locomotor activity, open field testing (OFT) was conducted in a polyvinyl chloride apparatus (SA215, SANS, Shenzhen, China; four equal zones 40 cm × 40 cm × 40 cm, central zone 30 × 30 cm). Mice were placed in the center and allowed to explore freely for 10 min while trajectories were captured using the ANY-Maze Video Tracking System (Stoelting, Wood Dale, IL, USA). Twenty-four hours later, cognitive performance was assessed using the novel object recognition test (NORT). Two identical objects were presented for 10 min during the exploration session. After a 2 h interval, one familiar object was replaced by a novel object, and mice explored for another 10 min. The recognition index (RI) was calculated as:

(1) $$RI=\frac{time2}{time1+time2}\times 100\%$$

where *time*1 and *time*2 represent the exploration time spent on the familiar and novel objects, respectively.

Tissue collection

After physiological and behavioral testing, all mice were euthanized six weeks post-modeling for blood and tissue collection. Blood samples were collected into EDTA-K2 anticoagulant and analyzed for complete blood counts using a BC-2800vet automated hematology analyzer (Mindray Inc., Shenzhen, China). The blood was then centrifuged at 3500× *g* for 15 min to separate serum. Tissues were collected using ceramic tools, weighed, and immediately processed for magnetic susceptibility, and then either frozen or fixed in 4% paraformaldehyde for subsequent histological and biochemical analyses.

### 2.3. Superconducting Quantum Interference Device (SQUID) Micro-Magnetometry

The magnetic properties of the samples were measured using a magnetic property measurement system 3 (MPMS3) SQUID magnetometer (MPMS3, Quantum Design, San Diego, CA, USA). Prior to the experiment, fresh mouse organs were weighed, placed in sample holders (8505-013(C130D), Quantum Design, USA), and vacuum-sealed. Magnetization–magnetic field (M-H) curves were acquired at 300 K over a field range of −10,000 Oe to 10,000 Oe, in 2500 Oe intervals. The magnetic susceptibility of each sample was calibrated using an empty holder. The magnetic moment of the sample was determined by subtracting the signal of the holder. The mass magnetization $\chi_{mass}$ was calculated as:

(2) $$\chi_{mass}=\frac{M}{\rho H}=\frac{\mu }{mH}$$

where *M* is the magnetization, *ρ* is the sample density, *μ* is the magnetic moment, *H* is the magnetic field strength, and *m* is the sample mass. For each measurement point, the magnetic moment was normalized by the sample mass to obtain *M*. All M-H data were linearly fitted using SquidLab in MATLAB R2022a, with the slope representing the magnetic susceptibility [14]. Four data points were collected at each nonzero field and six at the zero field.

### 2.4. Histology, Oxidative Stress Assays, and Iron Quantification

Histological analysis (H&E (hematoxylin and eosin) and oil red O)

Liver tissues were fixed in 4% paraformaldehyde, paraffin-embedded, and sectioned for H&E and Oil Red O staining. Images acquisition was performed using a light microscope, and quantitative histological metrics was performed using Fiji software (Fiji v2.14.0, National Institutes of Health (NIH), Bethesda, MD, USA).

Immunohistochemistry

To assess hypoxia-associated cellular response, immunohistochemical staining for hypoxia-inducible factor-1α (HIF-1α) was performed. Sections underwent deparaffinization, rehydration, endogenous peroxidase, antigen retrieval in sodium citrate buffer, and BSA blocking prior to primary antibody incubation (overnight, 4 °C). HRP-conjugated anti-rabbit IgG and Diaminobenzidine (DAB) chromogenic development were used for visualization, followed by hematoxylin nuclear counterstaining. The sections were then dehydrated and mounted, and the positive staining areas were quantified using Fiji software (NIH, USA). Imaging was performed using a light microscope.

Iron histochemistry

Iron deposition was visualized using DAB-enhanced Perls’ staining, which forms ferric ferrocyanide complexes that appear as intense blue-brown granules. The DAB amplification step improves signal contrast and detection sensitivity, allowing better correspondence with susceptibility-derived iron estimates from MRI. Quantification of staining intensity was performed using Fiji software (NIH, USA).

Ultrastructural imaging

Transmission electron microscopy (TEM) was used to assess iron-associated subcellular changes at nanometer resolution. Fixed tissue cubes were embedded in epoxy resin, sectioned (100 nm), stained with uranyl acetate, and imaged at 120 kV TEM (Tecnai G2 SPIRIT, FEI Corporation, Hillsboro, OR, USA). This allowed visualization of ferritin-like electron-dense granules and mitochondrial structural alterations, providing ultrastructural validation of iron.

Oxidative stress and apoptosis

To evaluate oxidative injury downstream of iron accumulation, ROS levels were assessed using dihydroethidium (DHE) staining, which fluoresces upon oxidation to ethidium and thus reports intracellular superoxide generation. Hepatocellular injury was further evaluated by TdT-mediated dUTP Nick-End labeling (TUNEL), which detects DNA fragmentation as a late-stage marker of apoptosis. Together, these assays capture both the upstream redox imbalance and its downstream cytotoxic consequences. Quantification was performed using mean DHE fluorescence intensity and the proportion of TUNEL-positive nuclei from at least three randomly selected fields per sample using Fiji software (NIH, USA).

Biochemical quantification of iron metabolism, lipid accumulation, and redox status

Serum aminotransferase, alanine aminotransferase, total bilirubin, total cholesterol, and low-density lipoprotein cholesterol (AST, ALT, TG, TC, and LDL-c) levels were measured to evaluate hepatocellular injury and lipid accumulation using an automated biochemical analyzer (Chemray 800, Rayto, Shenzhen, China). TG, TC, and LDL-c in frozen liver tissues were quantified to assess metabolic burden (A110-1-1, A111-1-1, and A113-1-1, Sinopharm, Beijing, China). Systemic activation of the iron-regulatory axis was evaluated by measuring serum erythropoietin (EPO) and ferritin levels using mouse ELISA kits (RXM2D2021166, Ruixinbio Quanzhou, Quanzhou, China and ELK10631, ELK Biotech, Wuhan, China, respectively). Hepatic lipid peroxidation was assessed by malondialdehyde (MDA) quantification (A003-1-2, Sinopharm, China), whereas superoxide dismutase (SOD) and glutathione (GSH) levels were measured as indices of antioxidant capacity (A001-3-2 and A006-2-1, Sinopharm, China). Total serum iron was measured using a commercial assay kit (A039-1-1, Sinopharm, China), while total iron and stable ferrous iron (Fe^2+^) in liver tissue were quantified by colorimetric assays and normalized to total protein content. In addition, bulk hepatic iron content was determined by inductively coupled plasma (ICP) to provide an absolute reference standard for tissue iron load.

### 2.5. Quantification and Statistical Analysis

No power analysis was performed; sample sizes were determined based on previous mouse studies [15,16]. To minimize bias, three independent researchers performed tissue preparation, imaging, and experimental procedures in a blinded manner. During data processing, only anonymized image data were used for quantitative analysis. Each experimental group included at least three mice. For each mouse, one tissue section was analyzed, and three randomly selected representative areas per section were quantified. Statistical analyses were performed using GraphPad Prism 9.0 (GraphPad Software, San Diego, CA, USA), and SQUID data fitting was conducted using SquidLab in MATLAB R2022a (MathWorks, Natick, MA, USA) [14]. For comparisons between two independent groups, a two-tailed unpaired Student’s t-test was used. For paired comparisons, a two-tailed paired Student’s *t*-test was applied. One-way ANOVA with Bonferroni post hoc correction was used for analyses involving three or more groups followed by multiple comparisons. Data are reported as mean ± standard error of the mean (SEM). Statistical significance was defined as *p* < 0.05.

## 3. Results

### 3.1. In Vivo MRI Characterization of Altitude-Associated Hepatic Magnetic Susceptibility Changes

**Figure 1:**
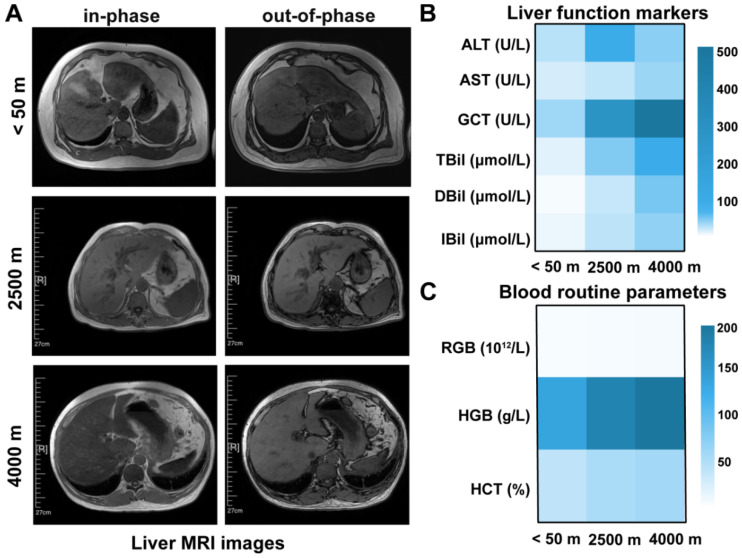
Hepatic MRI in-phase and out-of-phase imaging and biochemical alterations in individuals residing at different altitudes. (**A**) Representative in-phase and out-of-phase liver MRI images from male individuals (40–67 years) residing at different altitudes (<50 m, 2500 m, and 4000 m). (**B**) Liver function markers in individuals residing at different altitudes. (**C**) Blood routine parameters measured across the same altitude groups. Abbreviations: MRI, magnetic resonance imaging; ALT, alanine aminotransferase; AST, aspartate aminotransferase; GCT, glutamyl transferase; TBil, total bilirubin; DBil, direct bilirubin; IBil, indirect bilirubin; RBC, red blood cell; HGB, hemoglobin; HCT, hematocrit.

Liver MRI data were collected from six participants residing at high altitudes (2000–4500 m) and one participant living at low altitude (<50 m). The high-altitude cohort included one participant at 2000 m, two at 2500 m, one at 3800 m, one at 4000 m, and one at 4500 m. As shown in Figure 1A, the in-phase images from the 2500 m and 4000 m participants exhibited reduced signal intensity compared with their corresponding out-of-phase images and the low-altitude reference, indicating stronger susceptibility-related T2* effects. The low-altitude control displayed balanced in-phase and out-of-phase signal characteristics. Similar in-phase attenuation was also observed in the remaining high-altitude participants, although the magnitude varied across participants, with an in-phase hypointensity pattern observed in those living at 2000 m and 2500 m (Figure S1).

Figure 1B shows liver function markers from three representative participants across different altitudes showed varying degrees of hepatic impairment. Peripheral blood measurements further showed compensatory increases in red blood cell-related indices, especially hemoglobin and hematocrit, reflecting adaptation to hypoxic exposure (Figure 1C). Comparable hepatic and hematological abnormalities were detected among the remaining high-altitude participants (Figure S2A,B).

### 3.2. Establishment and Validation of a Simulated High-Altitude Mouse Model

**Figure 2:**
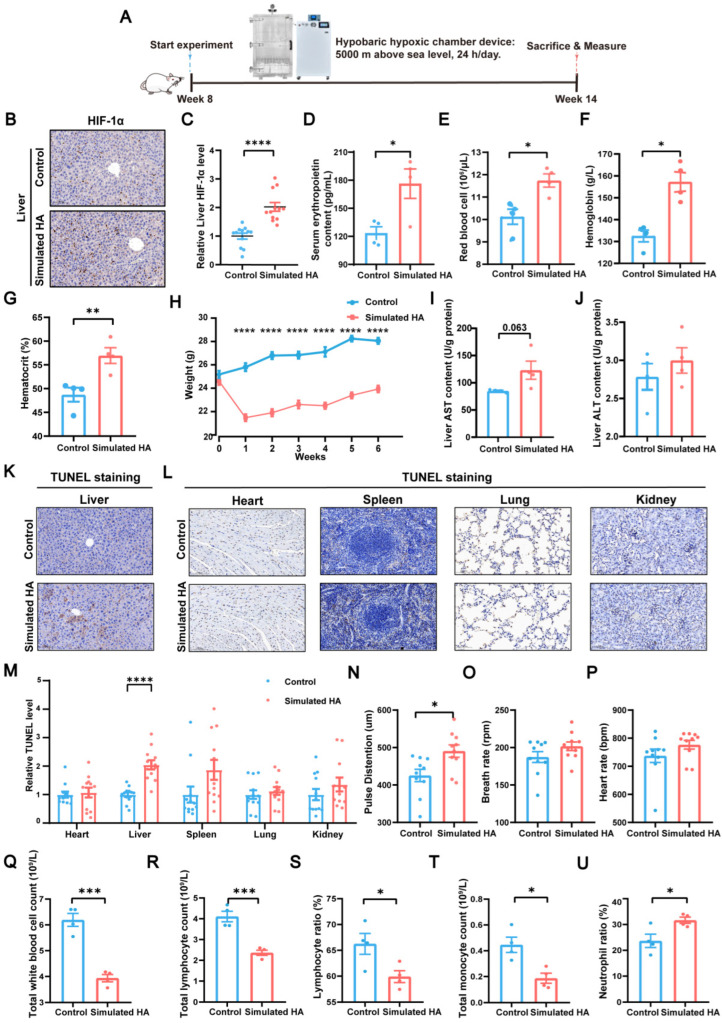
High-altitude condition induces hypoxic adaptation and liver injury in mice. (**A**) Experimental workflow for simulating high-altitude conditions in mice. (**B**,**C**) Representative hepatic HIF-1α immunostaining and quantification of positive regions (*p* < 0.0001, *n* = 4, 3 microregions per mouse, scale bar = 0.1 mm). (**D**) Serum erythropoietin levels (*p* = 0.02, *n* = 4). (**E**–**G**) Red blood cell count, hemoglobin levels and hematocrit (*p* = 0.02, *p* = 0.004, *p* < 0.01, respectively, *n* = 4). (**H**) Body weight (*p* < 0.0001, *n* = 10). (**I**,**J**) Liver AST and ALT levels (*n* = 4). (**K**–**M**) Representative TUNEL staining and quantification in the liver and major organs (*p* < 0.0001, *n* = 4, 3 microregions per mouse, scale bar = 0.1 mm). (**N**–**P**) Physiological parameters: pulse distention, respiratory rate, and heart rate (*p* = 0.01, *n* = 10). (**Q**–**U**) Total white blood cell count, total lymphocyte count, lymphocyte ratio, total monocyte count, and neutrophil ratio (*p* < 0.001, *p* < 0.001, *p* = 0.03, *p* = 0.01, *p* = 0.03, respectively, *n* = 4). Data are presented as mean ± standard error of the mean. * *p* < 0.05, ** *p* < 0.01, *** *p* < 0.001, **** *p* < 0.0001. Abbreviations: HA, high altitude; HIF, hypoxia-inducible factor; AST, aspartate aminotransferase; ALT, alanine aminotransferase; TUNEL, TdT-mediated dUTP Nick-End labeling.

To systematically investigate altitude-induced hepatic changes, we established a simulated high-altitude mouse model in a hypobaric hypoxic chamber reproducing the environment conditions of ~5000 m (Figure 2A). The hypoxic response was validated by significant upregulation of hepatic HIF-1α (*p* < 0.0001, Figure 2B,C) and elevated circulating EPO levels (*p* = 0.02, Figure 2D), leading to significantly increased hemoglobin concentration and hematocrit (*p* = 0.02, *p* = 0.004, *p* < 0.01, respectively, Figure 2E–G). Concurrently, high-altitude-exposed mice showed a significant reduction in body weight (*p* < 0.0001, Figure 2H) and a mild decrease in food intake (Figure S3A), reflecting typical responses to hypoxic stress.

Although HIF-mediated responses represent key adaptive mechanisms to hypoxia [17,18,19], chronic or severe exposure can exceed compensatory capacity and lead to organ injury [20,21]. In simulated high-altitude mice, hepatic dysfunction was indicated by elevated AST and ALT levels (Figure 2I,J). Systemic circulation also reflected liver dysfunction (Figure S3B–D). H&E staining showed mild vacuolar degeneration (Figure S3E), and TUNEL assays demonstrated an increase in hepatocellular apoptosis, with similar apoptotic patterns observed in the heart, spleen, lung, and kidney (Figure 2K–M).

Physiological monitoring revealed a significant increase in pulse vascular relaxation (*p* = 0.01, Figure 2N) and upward trends in both respiratory and heart rates (Figure 2O,P), indicating elevated cardiopulmonary workload. In addition, inflammatory indices including total white blood cell count, total lymphocyte count, lymphocyte ratio, total monocyte count, and monocyte ratio were significantly altered (*p* < 0.001, *p* < 0.001, *p* = 0.03, *p* = 0.01, *p* = 0.03, respectively, Figure 2Q–U), suggesting a systemic inflammatory and oxidative stress response. Behavioral assessments further showed hyperactivity in OFT and impaired recognition memory in the NORT (Figure S4). Taken together, these findings indicate that the simulated high-altitude condition induces multi-organ dysfunction, with the liver exhibiting particularly pronounced injury.

### 3.3. Magnetic Validation of Hepatic Susceptibility Changes in Simulated High-Altitude Mice

**Figure 3:**
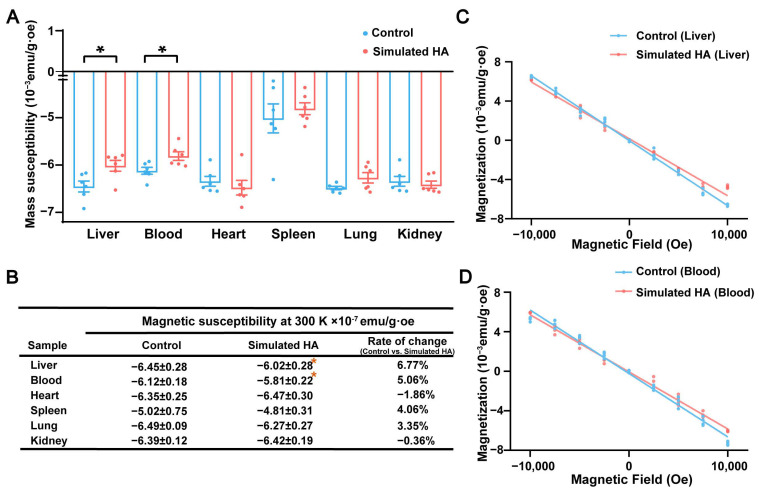
Increased liver magnetic susceptibility in simulated high-altitude mice. (**A**) SQUID-based measurement of tissue magnetic susceptibility in major organs at 300 K (*p* = 0.02, respectively, *n* = 6). (**B**) Quantification of magnetic susceptibility across organs (*n* = 6). (**C**,**D**) Representative M-H curves of liver and blood samples measured by SQUID at 300 K. Data are presented as mean ± standard error of the mean. * *p* < 0.05. Abbreviations: HA, high altitude; SQUID, superconducting quantum interference device; M-H, magnetization-field.

To verify whether hepatic magnetic susceptibility changes observed in human MRI were replicated in the animal model, SQUID micro-magnetometry was performed across organs. M-H curves acquired at 300 K revealed a significant increase in liver mass magnetization (*p* = 0.02) and an increase in blood mass magnetization (*p* = 0.02) in simulated HA mice, while the heart, spleen, lung, and kidney showed no significant differences between groups (Figure 3A–D). This organ-specific increase suggests localized accumulation of paramagnetic components.

The increased blood magnetization was consistent with elevated hemoglobin concentration, an adaptative erythropoietic response to hypoxia. In contrast, the significant elevation in liver magnetization likely reflects the hepatic accumulation of paramagnetic substances, particularly iron, given central role of liver in iron storage and metabolism. Despite localized increases, all tissues remained net diamagnetic overall, as expected from their high water content [22].

### 3.4. Hepatic Iron Overload as the Determinant of Magnetic Susceptibility Changes

**Figure 4:**
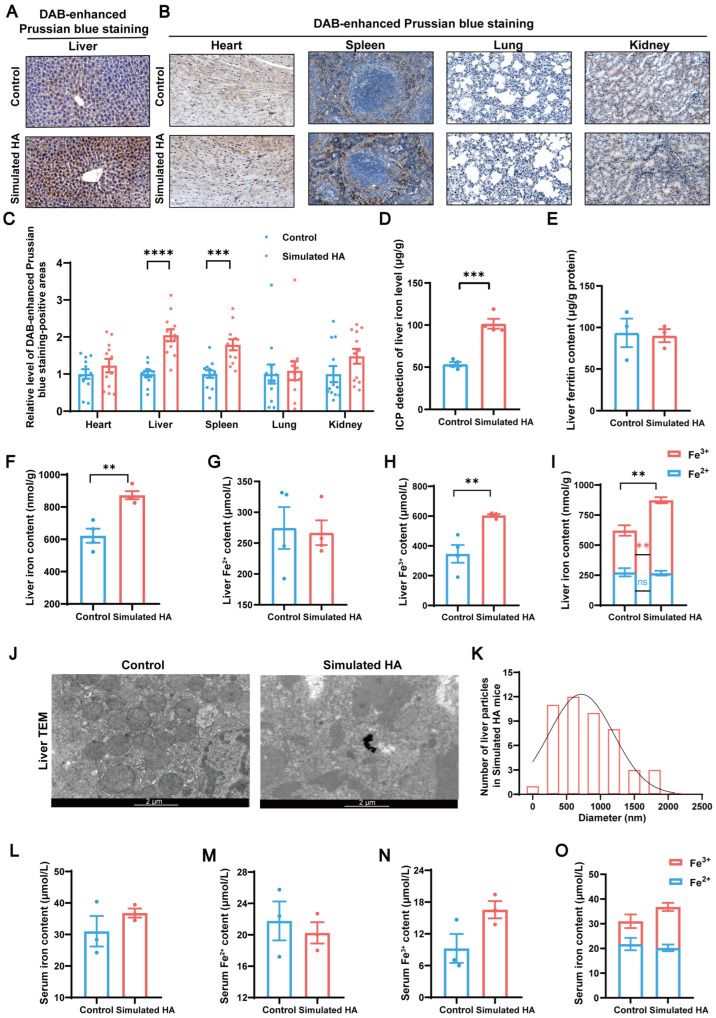
Iron overload in simulated high-altitude mice contributes to liver magnetic susceptibility changes. (**A**–**C**) DAB-enhanced Prussian blue staining of the liver and major organs, with quantification of iron-positive areas (*p* < 0.0001 and *p* < 0.001, respectively, *n* = 4 mice, 3 microregions per mouse, scale bar = 0.1 mm). (**D**) ICP-based measurement of total hepatic iron levels (*p* < 0.001, *n* = 4). (**E**) Ferritin levels in the liver (*n* = 3). (**F**–**I**) Iron, Fe^2+^, and Fe^3+^ levels in the liver (*p* = 0.002 and *p* = 0.005, respectively, *n* = 4). (**J**,**K**) Representative TEM images and particle size distribution of hepatic iron particles (*n* = 3, scale bar = 2 µm). (**L**–**O**) Iron, Fe^2+^, and Fe^3+^ levels in the systemic circulation (*n* = 3). Data are presented as mean ± standard error of the mean. ** *p* < 0.01, *** *p* < 0.001, **** *p* < 0.0001. ns, not significant. Abbreviations: DAB, 3,3′-Diaminobenzidine; HA, high altitude; ICP, inductively coupled plasma; TEM, transmission electron microscopy.

Given the established role of iron as a primary contributor to magnetic susceptibility [8] and central function of the liver in iron storage [5], we investigated hepatic iron metabolism under simulated high-altitude conditions. DAB-enhanced Perls’ staining revealed a significant increase in iron content in the liver (*p* < 0.0001) and spleen (*p* < 0.001) of simulated high-altitude mice, while other organs exhibited minimal changes (Figure 4A–C). Iron accumulation may explain the elevated liver magnetization in high-altitude conditions [23,24]. The spleen of simulated high-altitude mice likely showed only a trend toward increased magnetization due to the smaller increase in iron concentration. Quantitative analysis by ICP revealed an increase in total hepatic iron content in simulated high-altitude mice (*p* < 0.001, Figure 4D). Ferritin levels remained unchanged (Figure 4E), while total iron (*p* = 0.002) and ferric iron (Fe^3+^) (*p* = 0.005) were significantly elevated, with no significant change in Fe^2+^ levels, resulting in an increased Fe^3+^/Fe^2+^ ratio (Figure 4F–I).

The main factor driving the change in Fe^3+^ content in liver tissue is the variation in ferritin and hemosiderin content [25]. Since ferritin was unaffected, we suspected the iron was stored as hemosiderin. TEM further revealed large electron-dense iron aggregates (average diameter of 802.33 nm) in high-altitude mice livers but not in controls (Figure 4J,K). Based on the particle size and morphology, these aggregates were identified as hemosiderin-like deposits rather than ferritin particles [26,27]. Serum assays also showed a trend toward elevated total iron and Fe^3+^ levels in simulated high-altitude mice (Figure 4L–O). Together, these findings demonstrate that hypoxia-induced hepatic iron accumulation, particularly in the oxidized Fe^3+^ form, underlies the observed magnetic susceptibility changes.

### 3.5. High-Altitude Condition Induces Hepatic Lipid Accumulation and Oxidative Stress

**Figure 5:**
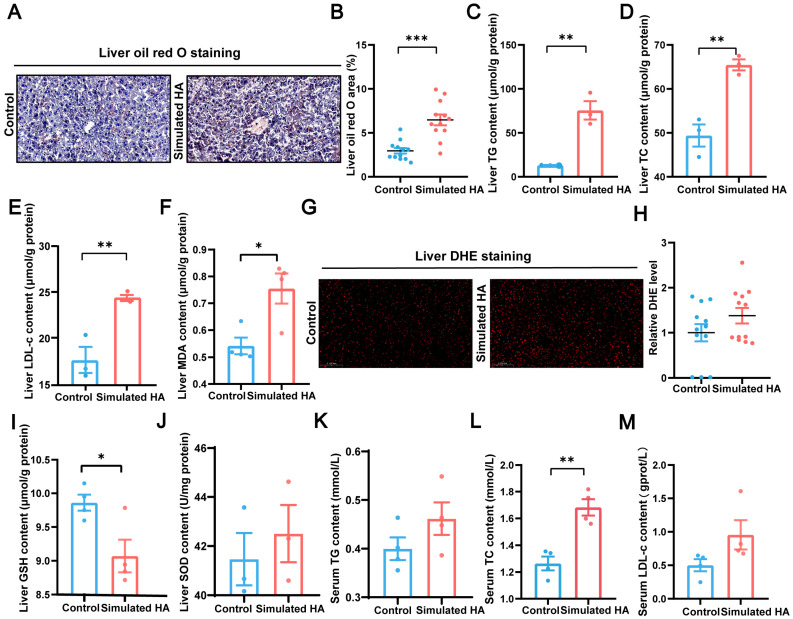
Lipid accumulation and oxidative stress in simulated high-altitude mice liver. (**A**,**B**) Representative Oil Red O staining and quantification of hepatic lipid accumulation (*p* < 0.001, *n* = 4 mice, 3 microregions per mouse, scale bar = 0.1 mm). (**C**–**E**) Hepatic levels of TG, TC, and LDL-c (*p* = 0.004, *p* = 0.005, *p* = 0.009, respectively, *n* = 3). (**F**) Hepatic MDA level (*p* = 0.02, *n* = 3). (**G**,**H**) Representative images and quantification of hepatic superoxide anion staining (n = 4, 3 microregions per mouse, scale bar = 0.1 mm). (**I**,**J**) Hepatic GSH and SOD levels (*p* = 0.02, *n* = 3). (**K**–**M**) Levels of TG, TC, and LDL-c in systemic circulation (*p* = 0.002, *n* = 3–4). Data are presented as mean ± standard error of the mean. * *p* < 0.05, ** *p* < 0.01, *** *p* < 0.001. Abbreviations: TG, triglyceride; TC, total cholesterol; LDL-c, low-density lipoprotein cholesterol; MDA, malondialdehyde; DHE, dihydroethidium; GSH, glutathione; SOD, superoxide dismutase.

Hepatic iron overload is closely associated with steatotic progression and plays a well-established pathogenic role in lipid degeneration and the development of insulin resistance [28]. Consistent with this, Oil Red O staining revealed a significant increase in hepatic lipid accumulation in simulated high-altitude mice (*p* < 0.001, Figure 5A,B). Biochemical analyses further confirmed disrupted lipid homeostasis, evidenced by elevated hepatic levels of TG, TC, and LDL-c (*p* = 0.004, *p* = 0.005, *p* = 0.009, respectively, Figure 5C–E). Serum lipid profiling also indicated systemic dyslipidemia, with significantly increased TC (*p* = 0.002) and rising trends in TG and LDL-c (Figure 5K–M). These findings demonstrate that simulated high-altitude exposure promotes hepatic lipid accumulation, likely as a downstream consequence of iron overload.

**Figure 6:**
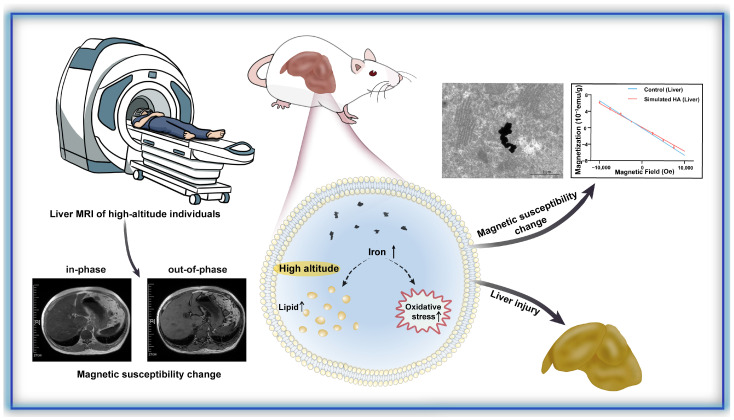
High-altitude exposure induces changes in liver magnetic properties in human and mice and causes functional impairment in mice.

In addition to lipid metabolic disruption, excessive iron can exacerbate oxidative stress by catalyzing the Fenton reaction, which converts Fe^2+^ to Fe^3+^ and generates excess ROS [29]. Correspondingly, MDA levels were significantly increased in high-altitude mouse livers (*p* = 0.02), indicating iron-induced lipid peroxidation (Figure 5F). DHE staining also revealed an increase in superoxide anion levels (Figure 5G,H). The oxidative stress response was further reflected by a significant decrease in GSH and an upward trend in SOD activity (*p* = 0.02, Figure 5I,J). Together, these data indicate that hepatic iron overload under simulated high-altitude conditions promotes oxidative injury through ROS generation and lipid peroxidation. Therefore, the significant increase in hepatic iron elevates magnetic susceptibility and simultaneously induces liver injury through lipid dysregulation and ROS-mediated oxidative damage (Figure 6).

## 4. Discussion

Our findings reveal a coordinated relationship between high-altitude hypoxic exposure and alterations in hepatic iron homeostasis, lipid metabolism, and redox balance. By integrating qualitative in vivo MRI observations from high-altitude residents with a controlled murine hypobaric hypoxia model, we demonstrate that exposure to high-altitude conditions is associated with hepatic iron accumulation and increased magnetic susceptibility. In the mouse model, quantitative ex vivo measurements confirmed an increase in hepatic iron content, supporting the interpretation that iron overload contributes to the observed susceptibility-related signal attenuation. Collectively, these findings suggest that hypoxia-induced iron dysregulation represents a key contributor to metabolic and oxidative liver dysfunction.

In the human cohort, dual-echo in-phase and out-of-phase MRI demonstrated relative signal attenuation patterns suggestive of compositional alterations in the liver. Signal attenuation on in-phase images may reflect increased susceptibility-related T2* decay, while signal loss on out-of-phase images relative to in-phase images is consistent with intravoxel fat–water signal cancellation and possible lipid accumulation [30]. Because gradient-echo MRI is sensitive to susceptibility-induced dephasing, reduced signal intensity may be compatible with increased paramagnetic content, including iron deposition [31,32]. However, the dual-echo protocol does not provide quantitative susceptibility or R2* estimation; therefore, the human MRI findings should be interpreted as qualitative and hypothesis-generating. Mechanistic support was provided by the murine model, in which SQUID magnetometry and ICP-MS confirmed increased hepatic susceptibility and iron burden.

Iron dysregulation is increasingly recognized as a driver of metabolic liver injury, contributing to steatotic progression, oxidative imbalance, and insulin resistance in nonalcoholic fatty liver disease [33,34]. In the context of hypoxia, iron accumulation may exacerbate oxidative stress through Fenton chemistry, in which Fe^2+^ is oxidized to Fe^3+^ with generation of reactive oxygen species that trigger lipid peroxidation and cellular injury [29]. Given the liver’s high metabolic activity and central role in detoxification, nutrient processing, and immunity regulation [35,36], it is particularly vulnerable to injury when exposed to physiologic stressors beyond its adaptive capacity [20,37,38]. Under chronic hypoxia, this vulnerability may be amplified, predisposing individuals at high altitude to iron-driven oxidative damage and metabolic compromise.

Hypoxia-inducible pathways may further link iron metabolism and lipid remodeling. Activation of HIF-1α has been reported to regulate iron uptake through transferrin receptor 1 (TFR1) axis and to promote ferroptosis-related cell death and liver injury [39,40]. In addition, hypoxia has been shown to simulate hepatic de novo lipogenesis and fatty acid uptake through HIF-1α and HIF-2α-dependent mechanisms [41]. At the same time, adaptive responses to chronic hypoxia may involve genetic regulation and behavioral plasticity that help maintain glucose–lipid homeostasis [42], potentially accompanied by epigenetic modifications and transcriptional reprogramming [43]. For example, transcriptomic studies in Tibetan and Leghorn chicken embryos incubated under hypoxic conditions have demonstrated upregulation of genes involved in fatty acid degradation, along with enrichment of transcription factors from the hepatic nuclear factor family that regulate lipid metabolism and energy homeostasis. These findings suggest that adaptive or maladaptive hepatic responses to hypoxia may involve multilayered regulatory mechanisms beyond acute oxygen sensing. Further investigation into epigenetic and transcriptional remodeling may provide deeper insight into the pathogenic processes underlying hepatic alterations at high altitude.

From a clinical perspective, susceptibility-related MRI findings in high-altitude populations should prompt careful assessment of iron distribution patterns. Chronic hypoxia stimulates erythropoiesis and systemic iron mobilization, potentially predisposing to secondary iron accumulation that is typically localized to the reticuloendothelial system, particularly the liver and spleen [44,45,46,47,48]. In contrast, iron deposition involving parenchymal organs such as the pancreas, heart, or pituitary is more suggestive of hereditary hemochromatosis [49], a distinction of clinical importance given that hypoxia-mediated hepcidin suppression may accelerate disease progression [44,50]. Accurate etiologic differentiation is essential for risk stratification, as myocardial iron deposition may aggravate high-altitude-related cardiac strain [51,52], while persistent hepatic iron overload may promote fibrosis and carcinogenic progression under sustained oxidative stress [53,54]. Importantly, magnetic susceptibility depends not only on total iron content but also on iron speciation and spatial organization [23]. In our study, although splenic iron content increased, splenic magnetic susceptibility showed only a nonsignificant trend. This discrepancy likely reflects microstructural compartmentalization of iron within reticuloendothelial macrophages, which can promote intravoxel field cancellation and attenuate bulk susceptibility changes despite elevated iron burden [50]. In contrast, more diffusely distributed hepatic iron aggregates may generate cumulative microscopic field perturbations that translate into measurable bulk susceptibility effects [23].

High-altitude adaptation may confer physiological resilience in certain contexts, including reduced postoperative pulmonary complications following liver resection [55], reflecting the dynamic balance between adaptive metabolic remodeling and organ-specific vulnerability under chronic hypoxia. Beyond diagnostic implications, our findings suggest potential applications in preventive and personalized medicine for high-altitude populations. Current preventive strategies, including acetazolamide prophylaxis, staged ascent, and oxygen supplementation [1,54], are typically applied without accounting for individual variability. The consistent detection of hepatic iron overload and magnetic susceptibility alterations raises the possibility that MRI-based assessment could facilitate risk stratification. Magnetic susceptibility and quantitative iron burden may serve as candidate biomarkers of altitude tolerance, helping identify individuals who may benefit from targeted interventions such as iron modulation therapies or antioxidant strategies. For long-term high-altitude residents, serial monitoring of these imaging markers may provide a means to assess cumulative physiological burden and support individualized prevention and early intervention before irreversible liver injury develops. Larger clinical studies are needed to clarify the long-term hepatic consequences of chronic high-altitude exposure and to better define the balance between adaptive and maladaptive mechanisms.

Our study has several limitations. First, although the hypobaric chamber effectively reproduces high-altitude hypoxia and reduced barometric pressure [13], the individual contributions of these components cannot be separated within this model. Second, both the clinical and experimental data were obtained at a limited number of discrete altitudes, future studies should systematically characterize dose–response relationships across a broader altitude spectrum. Third, given the preliminary nature of this study and the modest sample size, the dataset lacks sufficient statistical power for robust multivariate analyses. Nevertheless, the primary group-level comparisons consistently demonstrated statistically significant differences, supporting the reliability of the main findings. Several mechanistic issues also remain unresolved. The origin of excess hepatic iron, whether driven by increased intestinal absorption or splenic recycling of hemoglobin-derived iron [56], remains unresolved, and systemic regulators of iron homeostasis such as transferrin saturation and hepcidin were not assessed. Although hepatic total iron content and the Fe^3+^/Fe^2+^ ratio increased significantly while serum iron levels remained stable, this likely reflects the distinction between localized tissue iron storage and tightly regulated systemic iron pools under hypoxia-driven erythropoietic demand.

Beyond iron quantification, additional considerations merit discussion. Although iron is a primary determinant of tissue magnetic susceptibility [57], other paramagnetic substances, including Cu^2+^, Mg^2+^, and deoxyhemoglobin, may also influence magnetic properties. Direct assessment of these components would further strengthen mechanistic interpretation. Moreover, while this study focused on hepatic iron overload, the cognitive and behavioral abnormalities observed in high-altitude mice may reflect cerebral iron accumulation. Previous work has demonstrated that cerebral iron accumulation after high-altitude exposure [58], suggesting that future research should incorporate neuroimaging or histological evaluation of brain iron levels in hypoxia models. Finally, the identification of electron-dense particles as hemosiderin was based on morphology and Prussian blue staining [26,27], incorporating energy-dispersive X-ray spectroscopy or selected-area electron diffraction would provide definitive compositional and structural confirmation.

## 5. Conclusions

In summary, we demonstrate that sustained high-altitude exposure is associated with hepatic iron accumulation, increased magnetic susceptibility, and liver injury characterized by lipid deposition and oxidative stress. By integrating human imaging observations with controlled hypobaric hypoxia experiments, our findings link altitude-related hypoxia to iron-driven hepatic pathology. Beyond delineating a mechanistic pathway, this work provides a conceptual framework in which quantitative MRI metrics may serve as early indicators of hepatic vulnerability and inform individualized risk stratification and preventive strategies for populations residing at or travelling to high altitude, as well as for individuals exposed to hypoxic environments such as space missions.

## Data Availability

All data are available in the article and its Supplementary Files or from the corresponding author upon request.

## Supplementary Materials

The following supporting information can be downloaded at: https://www.mdpi.com/article/10.3390/biom16030353/s1. Supplementary figures S1–S4 and supplementary materials and methods are provided in a single document. Figure S1: Hepatic MRI in-phase and out-of-phase images of four other individuals living at high altitude. Figure S2: Liver function markers and blood routine parameters of four other high-altitude individuals. Figure S3: Functional impairments in simulated HA mice. Figure S4: Hyperactivity and non-spatial memory decline in simulated HA mice.
