# Supplementary material for: High-Altitude Condition Induces Hepatic Magnetic Susceptibility Changes and Liver Injury
Source: Biomolecules. 2026 Feb 26;16(3):353. doi: 10.3390/biom16030353 (PMC13023805; doi:10.3390/biom16030353)
Supplement: Supplementary file 1 [file biomolecules-16-00353-s001.zip › biomolecules-4153887-supplementary.pdf]

Supplementary Materials

High-altitude Condition Induces Hepatic Magnetic Susceptibility Changes and Liver Injury

Supplementary Figures

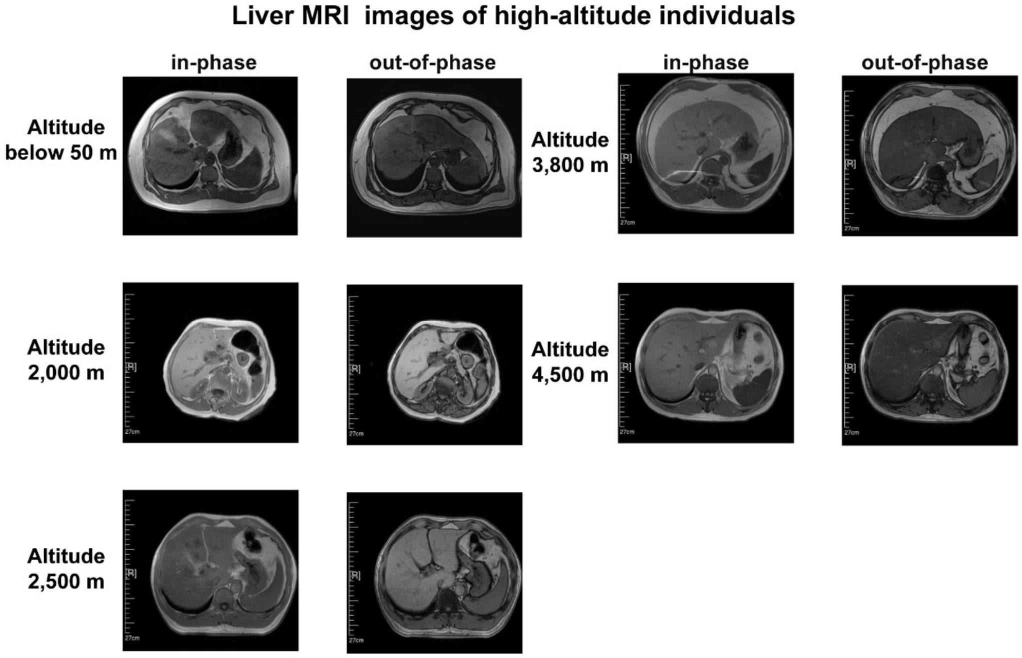

Fig. S1. Hepatic MRI in-phase and out-of-phase images of four other individuals live in high altitude, with altitudes of 2,000 m, 2,500 m, 3,800 m, and 4,500 m. The individual at an altitude of 2,000 meters is female, while the others are male. The age of the individuals ranges from 40 to 69 years.

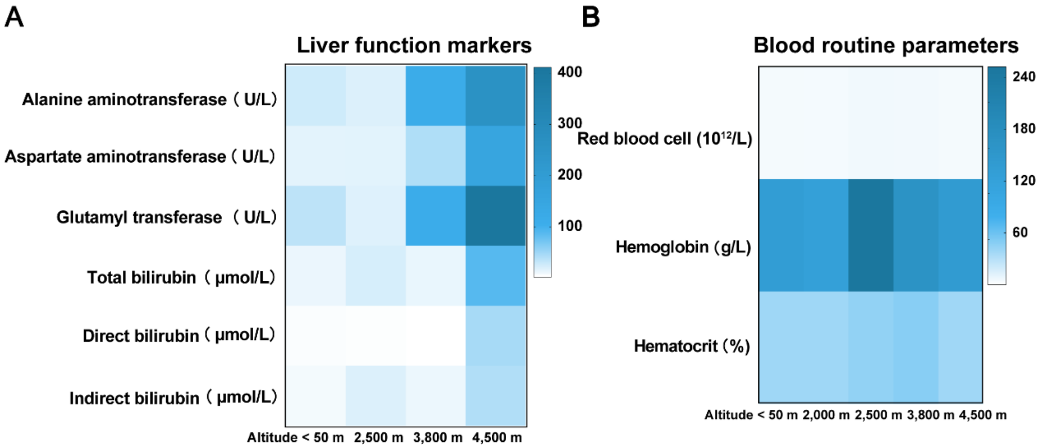

Fig. S2. (A) Liver function markers of four other high-altitude individuals. (B) Blood routine parameters of four other high-altitude individuals.

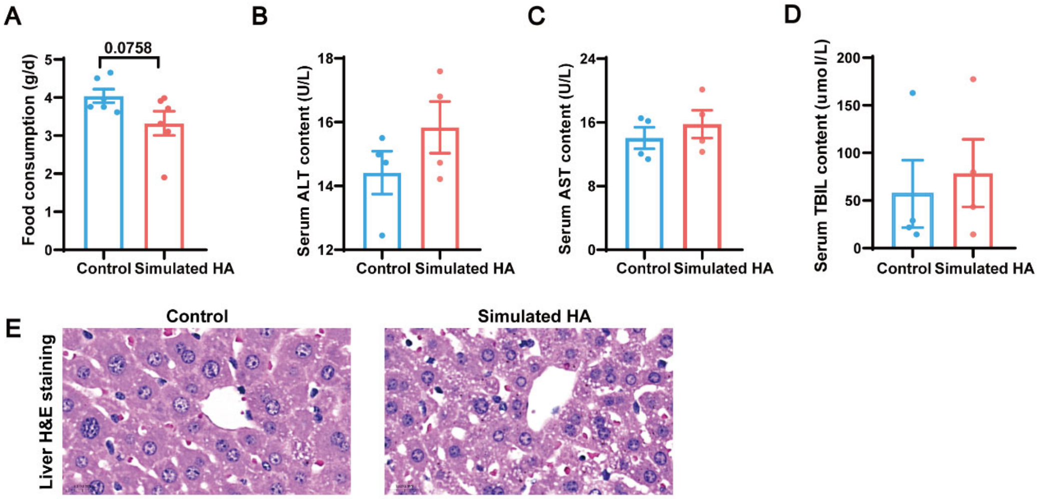

Fig. S3. Functional impairments in simulated HA mice. (A) Daily food intake of mice (n = 10 mice). (B to D) Levels of liver function markers ALT, AST, and TBIL in the systemic circulation (n = 4 mice). (E) H&E staining images of mouse liver. Data presented as mean ± SEM.

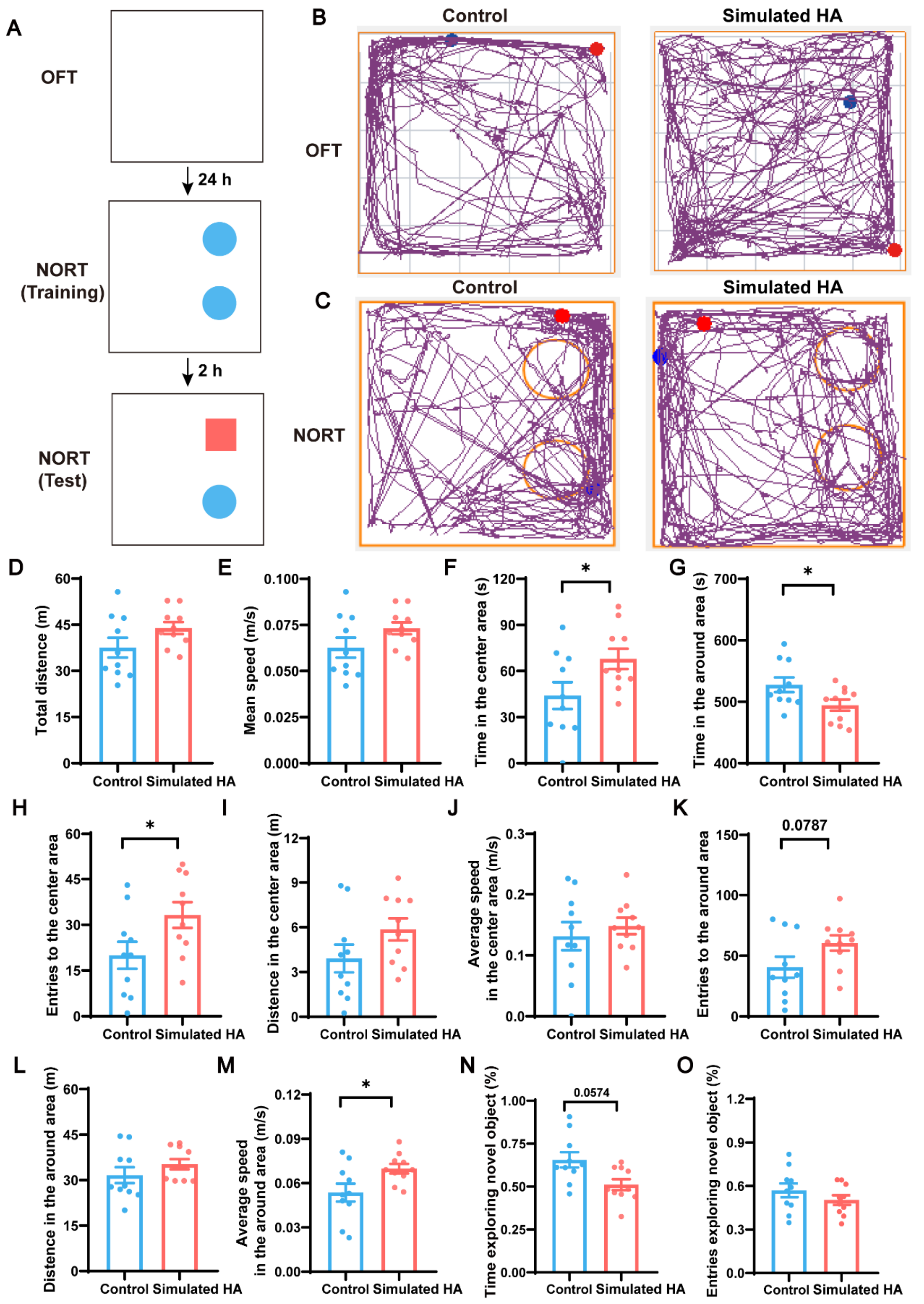

Fig. S4. Hyperactivity and non-spatial memory decline in simulated HA mice. (A) Illustration of OFT and NORT devices. (B) Representative mouse trajectory in the OFT. The blue and red dots show start and end points, respectively. (C) Representative mouse trajectory in the NORT. (D to G) Total distance traveled, average speed, time spent exploring the central area, and time spent exploring the around area in the OFT ($p = 0.042$, respectively, $n = 10$). (H to J) Movement parameters in the central area, including number of entries, distance traveled, and average speed in

the OFT ($p = 0.044$, $n = 10$). (K to M) Movement parameters in the around area, including number of entries, distance traveled, and average speed in the OFT ($p = 0.03$, $n = 10$). (N) Percentage of time spent exploring the novel objects in the NORT ($n = 10$). (O) Percentage of entries exploring the novel objects in the NORT ($n = 10$). Data presented as mean ± SEM. *$p < 0.05$.

Supplementary Materials and Methods

Immunohistochemical analysis

Liver tissues were fixed in 4% paraformaldehyde, dehydrated through a graded ethanol series, and embedded in paraffin. Paraffin-embedded samples were sectioned and subjected to immunohistochemical analysis. Tissue sections were treated with 3% H_2O_2, followed by antigen retrieval in 10 mmol/L sodium citrate buffer (pH 6.5). Non-specific antibody binding was blocked by incubation with 1% bovine serum albumin for 1 hour at room temperature. Sections were incubated overnight at 4°C with the primary antibody against HIF-1α (A11945, ABclonal, USA). After washing with TBST, sections were incubated at room temperature with HRP-conjugated goat anti-rabbit IgG secondary antibody (ab6721, Abcam, USA) for 1 hour. After DAB staining, sections were counterstained with hematoxylin, dehydrated, mounted, and the positive areas quantified using Fiji software (National Institutes of Health, USA). Imaging was performed using a microscope.

DAB-enhanced Prussian blue staining

Prussian blue reacted with iron in the tissue to form ferrous ferricyanide, clearly marking the distribution of iron. In addition, DAB (3,3′-diaminobenzidine) was used as a chromogenic substrate to further enhance the contrast and strength of the staining signal, increasing the sensitivity of iron detection. Specifically, tissues fixed in 4% paraformaldehyde were washed with PBS, sectioned, and incubated in freshly prepared Perls' solution (5% potassium ferrocyanide/10% HCl) for 1 hour. The sections were then incubated in DAB for 15 minutes. Quantification was performed using Fiji software (NIH, USA).

Dihydroethidium (DHE) staining

Frozen tissue sections (4-5 μm) were immersed in 1% acetone fixation solution at room temperature for 5 minutes, followed by PBS washing. Sections were then incubated with 10 μM DHE (309800, Sigma, USA) for 1 hour, washed with PBS, and imaged using a confocal microscope (SpinSR10, Olympus, Japan). The average fluorescence intensity of each image was quantified using Fiji software (NIH, USA).

TdT-mediated dUTP Nick-End labeling (TUNEL) staining

TUNEL assays were performed using a TUNEL assay kit (C1098, Beyotime, China) according to the manufacturer's instructions. Briefly, tissues were fixed with 4% paraformaldehyde and then embedded in paraffin. Paraffin-embedded tissue sections were deparaffinized and hydrated, followed by permeabilization with proteinase K. After incubating with 3% H_2O_2 at room temperature for 10 minutes, the sections were incubated with terminal deoxynucleotidyl transferase (TdT) reaction solution and incubated at 37°C in a humidified chamber for 60 minutes. Subsequently, the sections were incubated with streptavidin-HRP working solution at room temperature for 30 minutes. The slides were stained with DAB for 15 minutes at room temperature, followed by counterstaining with hematoxylin. After imaging, quantification was performed using Fiji software (NIH, USA).

Quantification of biochemical parameters (aminotransferase (AST), alanine aminotransferase (ALT), total bilirubin (TBIL), tissue triglycerides (TG), total cholesterol (TC), and low-density lipoprotein cholesterol (LDL-c))

Biochemical parameters including serum aspartate AST, ALT, and TBIL levels were measured using a biochemical analyzer (Chemray 800, Rayto, China).

Frozen liver tissue TG (A110-1-1, Sinopharm, China), TC (A111-1-1, Sinopharm, China), and LDL-c (A113-1-1, Sinopharm, China) were measured using commercial kits according to the manufacturer's instructions.

Erythropoietin (EPO) content measurement

The level of EPO in serum was measured using a mouse EPO kit (RXM2D2021166, Ruixinbio Quanzhou, China). A total of 50 μL mouse serum and biotinylated antibody working solution were added to the pre-coated wells of a microplate and incubated in the dark at 37℃ for 30 minutes. After washing, HRP-conjugated working solution was added, and the plate was incubated in the dark at 37℃ for 30 minutes. Following another wash, 100 μL substrate solution was added, and the plate was incubated in the dark at 37℃ for 30 minutes. Finally, 50 μL stop solution was added to each well, and the absorbance (OD value) was immediately measured at 450 nm. The EPO concentration in the serum was calculated based on the OD values.

Ferritin level measurement

Ferritin levels in mouse liver were quantified using an ELISA kit (ELK10631, ELK Biotech, China) according to the manufacturer's instructions. Briefly, liver tissue was homogenized, and standards or samples were added to wells pre-coated with detection antibodies. The plates were incubated at 37°C for 60 minutes. After discarding the liquid and washing six times, 100 μL of HRP-labeled streptavidin was added and incubated for 30 minutes. Substrate solution (TMB) was added, and the plate was incubated at 37°C in the dark for 15 minutes. Finally, the termination solution was added, and absorbance was measured at 450 nm using a microplate reader. Data were further normalized to the total protein content of the liver tissue.

Malondialdehyde (MDA) level measurement

Liver MDA levels were measured using an MDA assay kit (A003-1-2, Sinopharm, China). Tissue was homogenized and lysed with tissue lysis buffer. The mixture was centrifuged at 12,000×g for 10 minutes at 4°C, and the supernatant was collected. TBA dilution was prepared by dissolving 1.85 mg of TBA in 500 μL of buffer and vortexing at 70°C in the dark to aid dissolution. The solution was mixed with 30 μL of antioxidant and diluted to a final volume of 1.5 mL with water to make the TBA working solution. Next, 250 μL of tissue homogenate and 500 μL of TBA working solution were mixed and boiled for 15 minutes. After cooling to room temperature, the mixture was centrifuged at 1,000×g for 10 minutes, and the supernatant was transferred to a 96-well plate. Absorbance was measured at 532 nm using a microplate reader, and data were further normalized to the total protein content of the liver.

Superoxide dismutase (SOD) and glutathione (GSH) levels measurement

SOD levels in liver tissue were measured using an SOD assay kit (A001-3-2, Sinopharm, China), and GSH levels were measured using a GSH assay kit (A006-2-1, Sinopharm, China), according to the manufacturer's instructions. Data were further normalized to the total protein content of the liver.

Total iron and Fe^{2+} level measurement

Total serum iron levels were measured using a serum iron assay kit (A039-1-1, Sinopharm, China) according to the manufacturer's instructions. For tissue analysis, total iron levels in liver tissue were measured using a total iron assay kit (A039-2-1, Sinopharm, China), and Fe^{2+} levels were measured using an Fe^{2+} assay kit (AKIC004 M, BOXBIO, China). Tissue total iron and Fe^{2+} contents were then normalized to protein content.
